# The Role of Working Memory in Age-Related Emotional Memory Bias

**DOI:** 10.1007/s42761-022-00134-5

**Published:** 2022-08-31

**Authors:** Negin Sattari, Lauren N. Whitehurst, Sara C. Mednick

**Affiliations:** 1 Department of Psychiatry & Human Behavior, University of California, Irvine, CA USA; 2 Department of Psychology, University of Kentucky, Lexington, KY USA; 3 Department of Cognitive Science, University of California, Irvine, CA USA

**Keywords:** Aging, Working memory, Long-term memory, Emotional bias

## Abstract

Aging is accompanied by deterioration in both working memory (WM) and long-term memory (LTM), yet whether these changes are related is not understood. Sleep plays a role in the formation of LTM in young adults, but the findings in older adults are not as clear. The types of memories we store also shift with age as young adults preserve a higher proportion of negative experiences when compared to older adults. The reason for this age-related change in emotional memory bias is also not clear; however, some studies have suggested that WM changes across aging may be an important factor. In the current study, we examined performance in WM and emotional LTM in younger and older adults. We added a daytime nap in half the subjects to examine a possible role of sleep on emotional LTM. In the morning, 93 younger (18–39) and 121 older (60–85) adults completed a WM task. Subjects also encoded neutral or negative word pairs and provided valence and arousal ratings for each pair. After half the subjects took a daytime nap, LTM was examined, and valence and arousal ratings were reassessed. Results indicate that older adults showed worse recognition for negative word pairs compared with neutral, as well as decreased negative valence ratings in the afternoon. This decrease in emotional reactivity was correlated with better LTM performance. In contrast, younger adults performed better on the negative compared to neutral word pairs, with no change in emotional reactivity and no association between emotional reactivity and LTM. In addition, WM was positively related to LTM in younger, but not in older adults. Lastly, no differences were shown across sleep, regardless of age. Our findings suggest that the emotional memory bias may be associated with the emotional saliency of the information in older adults, and with WM capacity in younger adults.

**Supplementary Information:**

The online version contains supplementary material available at 10.1007/s42761-022-00134-5.

Aging is associated with declines in long-term memory (LTM) maintenance, with older adults often having worse LTM performance compared to younger adults. A range of cognitive and physiological factors, including working memory (WM; i.e., the ability to hold information in mind for a short period; Baddeley, 2010), emotionality (i.e., valence; Otani et al., 2007), and sleep (Van der Helm & Walker, 2011), may play a role as each of these dynamics have been shown to facilitate LTM formation in younger adults. However, the unique roles these cognitive and physiological factors play in LTM maintenance across aging are less clear. In the current study, we examine the modulatory role of WM capacity on an emotional LTM task after either a daytime nap or a period of wakefulness in younger and older adults.

Older adults frequently describe deterioration in memory (Hohman et al., 2011), and objective measures validate these reports with decreases in both LTM and WM across the lifespan (for review, see Reid & MacLullich, 2006). Specifically, declines in performance have been shown in hippocampus-dependent, episodic memory (Miller & O’callaghan, 2005; Park & Reuter-Lorenz, 2009), spatial navigation (Barnes, 1979; Gage et al., 1984; Markowska et al., 1989), and associative learning (Behrends et al., 2007). Importantly, associations between WM performance and LTM maintenance have been found. Lugtmeijer et al. (2019) tested both younger and older adults on WM and episodic LTM tasks and found that WM and LTM were positively related, but only among younger adults. In addition, a series of studies have reported an overlap between brain regions associated with WM and LTM processing among 19 to 40 years old subjects (Ranganath et al., 2003), specifically in the pre-frontal cortex (PFC; Jetter et al., 1986; McAndrews & Milner, 1991; Eslinger & Grattan 1994; Moscovitch & Winocur 1995). Together, these studies suggest that WM and LTM processing are intimately related and declines in LTM performance among older adults may be associated with a similar decline in WM ability.

Along with memory capacity losses that often accompany aging, negative memory biases also shift with aging. Younger, compared to older adults, have better memory for negatively valenced information. This negative memory bias is likely influenced by physiological systems that are tuned to organismal survival and enhance LTM formation through the release of memory-modulating neurohormones, like cortisol and epinephrine. This idea is supported, in part, by the stress-dependent enhancements of memories in younger adults, but often not older adults, when acute stress is experienced peri-encoding (Gagnon & Wagner, 2016; Sazma et al., 2019; Smith et al., 2019; See meta-analysis: Shields et al., 2017). Some theories suggest that the lack of negative memory biases in older adults may be due to life experience and changing priorities with aging. Specifically, the socioemotional selectivity theory posits that as we age, motivational goals associated with emotional well-being become increasingly salient as perceived age-related time limitations come into greater focus (Carstensen et al., 1999). These motivational goals shape top-down attentional dynamics and lead to greater retention of positive information. It is important to note however that motivational goals may not be the only factor influencing this age-related negative memory bias. In fact, WM dynamics may also play a role here. One study measured older adults’ LTM for positive and negative pictures as well as their WM and reported that older adults with superior WM recalled a higher proportion of positive pictures and older participants with inferior WM recalled fewer positive pictures (Mather & Knight, 2005). These findings suggest that emotion and WM may each be associated with LTM performance and related to the reasons older adults’ emotional memory biases exist (Mather & Knight, 2005).

Another potential candidate for memory modulation across the lifespan is sleep. It has been shown that sleep benefits memory in young adults (Rasch & Born, 2013; Stickgold, 2005), with this effect particularly pronounced for negatively valenced representations (Lipinska et al., 2019; Mather & Knight, 2005, meta-analysis). Both sleep quality and quantity decline across aging (Pace-Schott & Spencer, 2011; Dijk et al., 2010) and these changes may contribute to the shifting role of sleep for memory with aging (Sattari et al., 2019; Scullin, 2011). Jones et al. (2018) showed that recognition of negative and neutral pictures was not different between sleep and wake groups among middle-aged subjects. In another study, the same researchers reported preserved, but selective, sleep-dependent memory processing among older adults, in which older adults, compared to younger adults, showed better memory for positive stimuli after sleep. This effect is aligned with the socioemotional selectivity theory and further suggests that sleep may work to further bias memory representations towards emotional well-being for older adults (Jones et al., 2016). Taken together, this suggests there is an age-related change in the relative influence of sleep compared to wake on emotional memory.

The goal of the current study is to investigate the roles WM, sleep, and emotionality play in long-term memory formation in young and older adults. We tested a group of older and younger subjects on both WM and LTM in the morning. After finishing the cognitive tasks, half of the subjects took a daytime nap to see if sleeping between encoding and test would amplify the emotional memory bias. In the afternoon, all subjects took a recognition memory test for the word pairs. We predicted that older adults would show worse memory for negative word pairs, compared with neutral pairs, whereas younger adults would show better memory for the negative compared to neutral word pairs. We expected this effect to be exacerbated for those who took a nap. We also tested emotional reactivity (measured by change in valence and arousal rating) during encoding and recognition, to test the prediction that emotional reactivity would be associated with the emotional memory bias. Lastly, we predicted that WM will be correlated with both negative and neutral word pair memory in younger, but not older adults. We also expected these correlations would be stronger after the nap for younger adults.

## Method

### Subjects

Ninety-three younger adults (41 females) between the ages of 18 and 39 (M = 21.02, SD = 2.91) and 121 older adults (58 females) ages of 60–85 (M = 72.5, SD = 6.05) gave informed consent to participate in the study. Inclusion criteria required adults to be healthy, non-smoking adults with a regular sleep schedule (defined as 6 to 9 h of sleep per night on average). Subjects had no personal history of sleep, neurological or psychological disorders, or other chronic illnesses. Medical history was assessed twice. First, a general health condition report was collected over the phone (or internet survey) during pre-screening, and eligibility was determined. Second, at orientation, older subjects were given details about the study and screened for cognitive ability using the Digit Span Backwards (DSB) and dementia using Telephone Screening for Dementia questionnaires (referred to as TELE; Gatz et al., 2002), respectively. The DSB task contains a varying length of digit strings that subjects were asked to repeat backwards after it was read to them. All the experimental procedures were approved by the Institutional Review Board of the University of California, Riverside.

Eligible subjects were asked to maintain a regular sleep schedule (6–9 h per night) for 1 week prior to their visit, which was monitored with sleep diaries. In addition, subjects were asked to wear an actigraph (Actiwatch Spectrum, Respironics) for one night prior to their visit to ensure they received adequate sleep. Subjects were rescheduled if they reported poor sleep quality in their sleep diary, such as having more than 2 nights of sleep that was less than 6 h during the week prior to their visit, or if subjects’ actigraphy data detailed less than 6 h of sleep the night before the experimental visit. If subjects’ sleep did not meet this criterion, they were given another week to fill out a new sleep diary and maintain a regular sleep/wake schedule prior to their visit. Subjects were asked to refrain from consuming caffeine, alcohol, and all stimulants for 24 h prior to and including the study day.

### Protocol

**Fig. 1:**
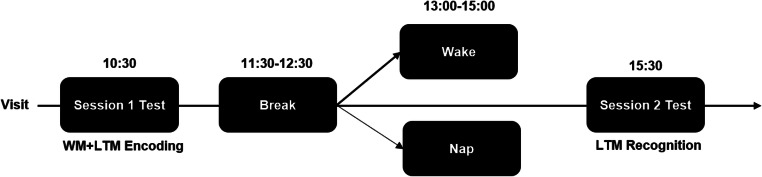
Subjects were tested on a WM task and encoded word pairs in the morning at session 1. Task order was counterbalanced. After the break, subjects were randomly assigned to either take a nap or stay awake. At session 2, subject’s LTM was tested in a recognition task

Subjects arrived at the Sleep and Cognition (SaC) lab at 11 a.m. for session 1. WM was tested using the operation span task (OSPAN). A word pair associates task with either two neutral or one negative and one neutral word was used for LTM. These words were encoded during session 1 and emotional reactivity (valence and arousal) for each word pair was provided by the participant (Fig. 1). Task order was counterbalanced across subjects and within sleep/wake group. At 12:30 p.m., subjects were randomly assigned to either a nap or a wake condition. For the nap, subjects were provided a 2-h nap opportunity and were allowed to sleep up to 90 min. In the wake group, subjects could remain in the lab and engage a range of activities from playing board games with a lab research assistant, watching TV, reading in the lab, or they were permitted to leave and return for session 2. All wake subjects were instructed to abstain from caffeine, exercise, and napping during their break and were asked to report back to the lab at 3:30 p.m. for session 2. Subjects’ activity levels were also tracked with actigraphy during their breaks to ensure they did not nap. During session 2, subjects completed recognition for the word pair task and again rated the valence and arousal of each word pair.

### Cognitive Tasks

The cognitive tasks we utilized in this project were designed for an event-related potential (ERP) study for which the data are not yet published. As such, the timing of the stimuli presentation and testing protocol were created for that purpose. Due to timing issues related to the ERP design, specifically, the long-presentation times likelihood to induce ceiling performance effects, we did not include an immediate test for the long-term memory WPA task. Detailed descriptions of each task are below.

### WPA Task

**Fig. 2:**
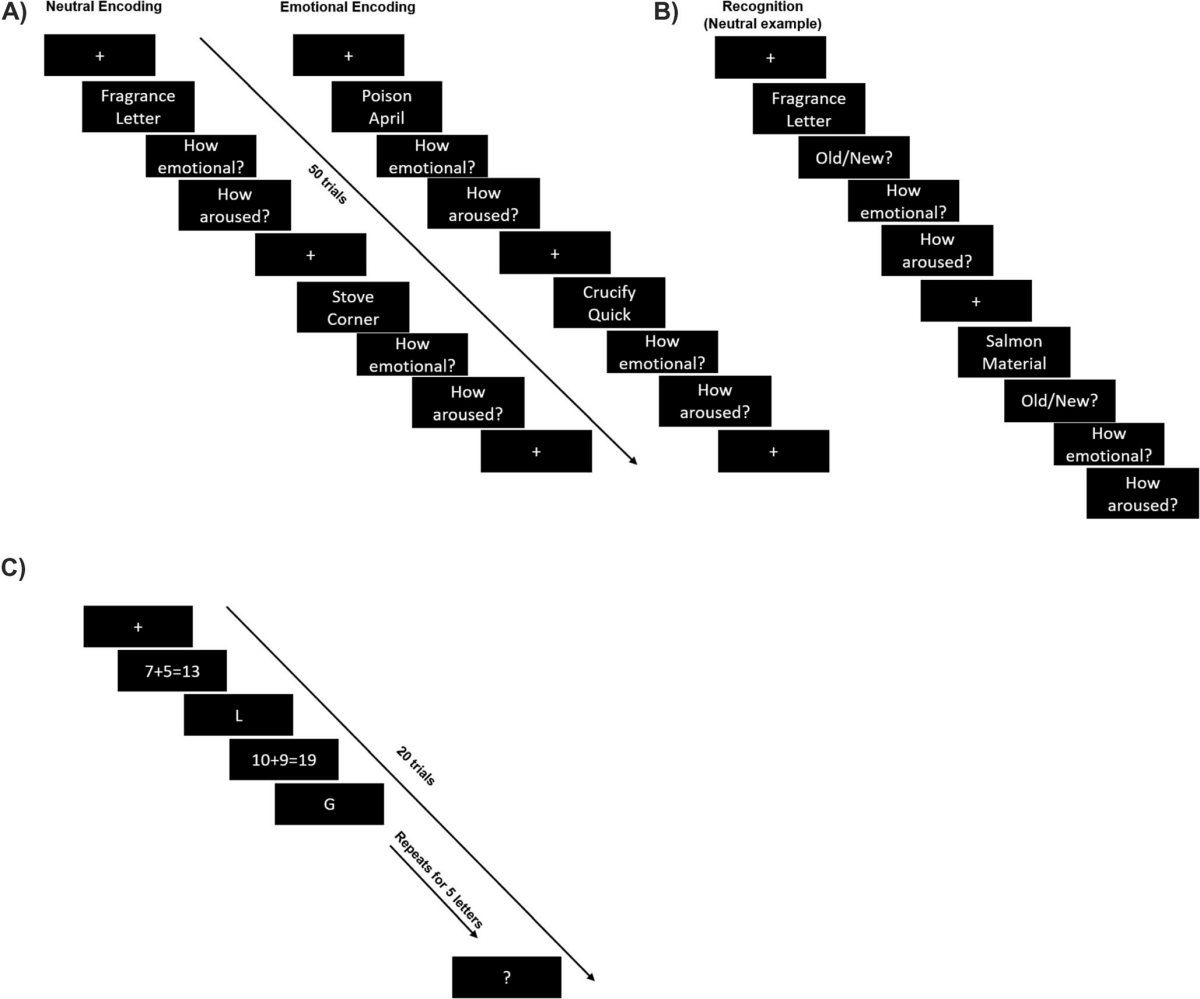
Memory tasks. **a** WPA encoding for both neutral and emotional word pairs. **b** WPA recognition test for the neutral condition. **c** WM task

Words were chosen from the Affective Norms for English Words (Bradley & Lang, 1999). During encoding, all subjects viewed 50 unrelated, word pairs on a computer monitor. Sequential word pairs were presented to participants. Each word was on the screen for 1,000 ms followed by a blank screen with a fixation point for 2,000 ms. After each pair presentation, participants were asked to rate arousal and valence of the word pairs. Valence was rated on a scale of one to seven with one being very negative, four being neutral, and seven being very positive. Arousal was rated on the same scale; one was low and seven was high arousal. For this task, words in each pair were either both neutral valences or one of the words was negatively valenced (negative word was always presented in top position on computer screen; see Fig. 2a). Thus, each subject only encoded emotional pairs or neutral pairs. During session 2, after the sleep intervention, subjects were given a recognition task with all the word pairs from session 1 and 25 new pairs and were asked to determine if each pair was new or old. They were then asked to rate the level of arousal and valence again (see Fig. 2a, b).

### Working Memory Task (OSPAN)

At session 1, subjects were tested on 20 trials of the operation span task (adapted from Lewandowsky et al., 2010). Subjects viewed a serial visual display of letters and math problems. They were asked to hold the letters in memory while simultaneously determining if the simple math problems were correct (e.g., 7 + 5 = 13). Based on pilot data, we selected letter strings that would equate performance differences between younger and older subjects. For older adults, the letter strings were between 3 and 5 letters long, and for youngers, the strings were 4 to 6 letters long (see Fig. 2c). Each math equation was on the screen for 3,000 ms, and each letter was presented for 1,300 ms. During the trial, subjects were instructed to press designated keys on the keyboard to indicate equation accuracy. At the end of each trial, a question mark appeared on the screen to signal the participant’s response. Subjects were required to enter the letters in the same order they were presented. Subjects’ data were only included in the analysis if they performed equal and/or greater than 75% correct on the math portion. Recall was not timed and subjects were not able to go to the next trial until the correct number of letters was entered. Four breaks were equally spaced throughout the task. All subjects were provided three test trials at the start of the task to ensure task comprehension. To this end, some subjects required extra practice trials. The practice trials were not counted towards the subjects’ final score.

### Data Reduction

In this study, if subjects were not comfortable during any part of the experimental day, they were compensated for the portion that was completed and were exempt from finishing the day (among the older subjects, 2 decided to drop out after the encoding session). In addition, for sleep analysis, 5 subjects (2 older and 3 younger adults) were not included due to missing data because of technical computer problems. Further, one younger and two older adults were excluded from the WM analysis due to not meeting the math criterion.

### Electroencephalography (EEG)

EEG data were acquired using a 32-channel cap (EASEYCAP GmbH) with Ag/AgCI electrodes placed according to the international 10–20 system (Jasper, 1958). Electrodes included 24 scalp, two electrocardiogram (ECG), two electromyogram (EMG), two electrooculogram (EOG), 1 ground, and 1 on-line common reference channel. EEG signals were recorded at a 1000 Hz sampling rate and referenced on-line to the common reference channel. Next, EEG signals were re-referenced. The electrodes on the right side of the scalp were re-referenced to the left mastoid and the electrodes from the left side of the scalp were re-referenced to the right mastoids. Sleep architecture variables included minutes and percentage of stage 1, stage 2, slow wave sleep (SWS), and rapid eye movement (REM), as well as total sleep time (TST), sleep latency (SL), wake after sleep onset (WASO), and sleep efficiency (SE).

### Statistical Analysis

The data presented are part of a study that recorded event-related potentials during working and long-term memory. As such, the power analysis that was conducted to determine participant sample size was aligned with the variables observed for the ERP study design and was not conducted for the statistical analyses presented here.

Memory performance for the WPA at session 2 was assessed by calculating the following variables: hit rate, false alarm rate, and dprime (Zhit rate − Zfalse alarm rate, where Z is the inverse of the cumulative distribution function and a measure of memory discriminability). We used a multivariate analysis of variance (ANOVA) with memory measures (e.g., dprime) as the dependent variable and sleep, age, and word-type (emotional, neutral) as the fixed factors. In addition, we examined the change in arousal and valence rating in a difference score (session 2 − session 1). We included these variables in a univariate ANOVA with the dependent variables arousal change and valence change and fixed factors sleep, age, and word-type. Further, Student’s *t-*test was used for comparisons within each group. Next, one-sample *t*-tests were used to compare change in arousal and valance across the day to zero (no change) in each group. WM capacity was assessed by calculating the accuracy (total number of correct recalls). The accuracy for WM performance was averaged across the three difficulty levels (i.e., 3, 4, and 5 letter length) for bivariate correlation tests. Finally, we used bivariate correlations to assess the relationship between WPA performance and OSPAN as well as performance with arousal and valence change.

## Results

### Sleep Architecture

Sleep architecture can be found in Table 1. In general, and consistent with the previous findings, compared to younger adults, older adults experienced lighter and more fragmented sleep. Specifically, older adults sleep had more stage 1, S1(%):*t*(92) = −2.55, *p* = .01, less SWS, *t*(94) = 3.93, *p* < .001; SWS(%):*t*(94) = 3.27, *p* = .001, and less REM, REM:*t*(94) = 2.36, *p* = .002; REM(%):*t*(94) = 2.31, *p* = .02. Older adults also woke up more frequently after falling asleep and had lower sleep efficiencies, WASO:*t*(88) = −4.84, *p* < .001; SE:*t*(91) = 5.51, *p* < .001 as well as lower sleep durations overall, TST:*t*(91) = 3.29, *p* = .001.

**Table 1:** Sleep summaries during nap

Sleep stages	S1	S2	SWS*	REM*	S1(%)*	S2(%)	SWS(%)*	REM(%)*	WASO*	SE(%)*	TST*
Younger adults	4.15 (0.44)	29.88 (2.01)	19.29 (2.07)	5.81 (1.12)	9.39 (1.84)	52.65 (2.92)	30.15 (2.97)	7.79 (1.42)	6.33 (0.96)	76.85 (2.44)	60.27 (3.15)
Older adults	5.66 (0.74)	25.87 (2.46)	8.62 (1.58)	2.31 (0.87)	19.51 (3.76)	58.44 (3.71)	16.33 (2.90)	3.32 (1.22)	17.18 (2.30)	54.39 (3.38)	44.54 (3.50)

Notes: For both younger adults (first row) and older adults (second row), data are reported as mean (SEM). *REM* rapid eye movement, *SE* sleep efficiency, *SWS* slow wave sleep, *WASO* wake after sleep onset, *TST* total sleep time. Significant differences are shown in bold and * next to the column name represents *p*-value < .05

### Nap vs Wake on LTM Performance

**Fig. 3:**
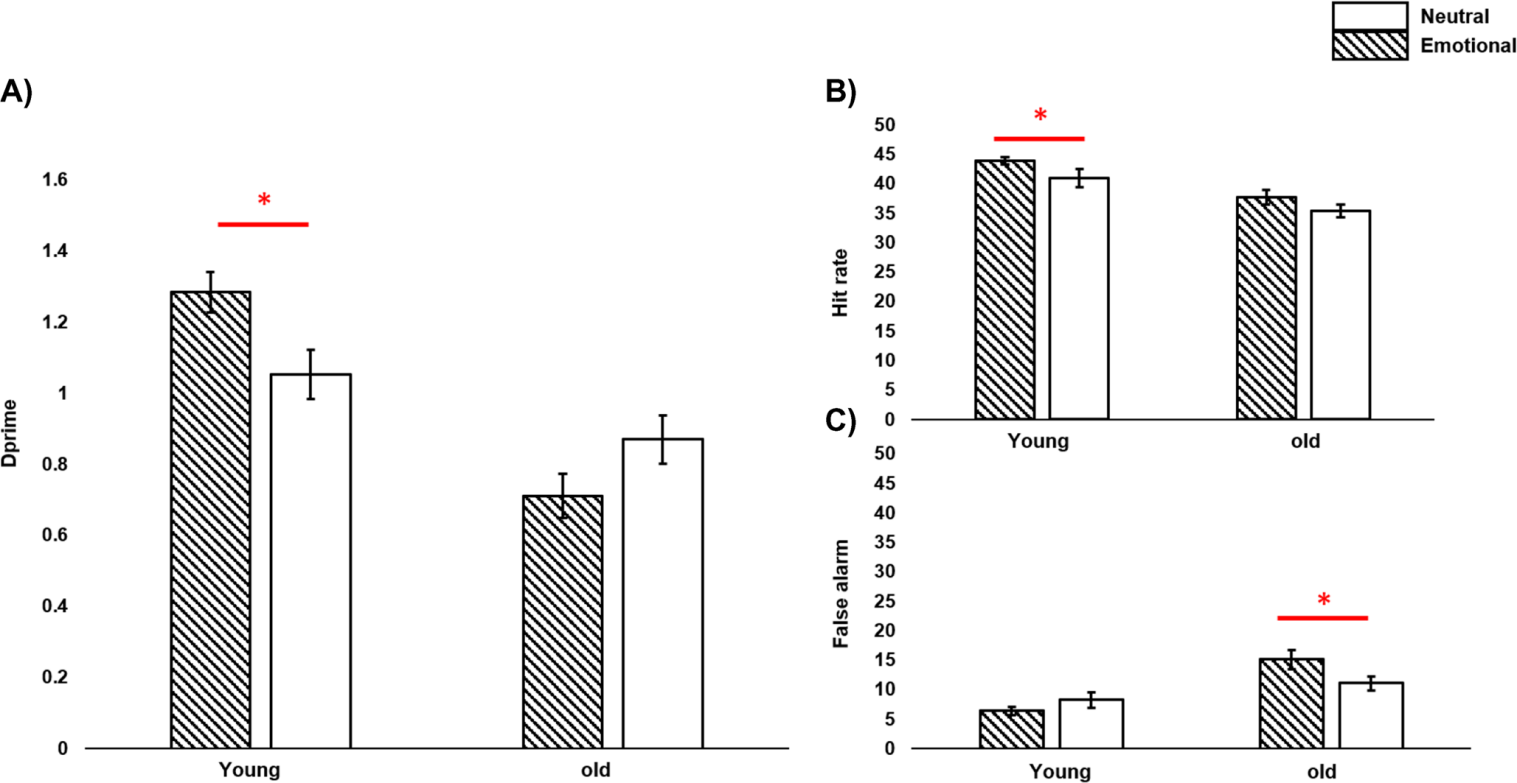
Long-term memory measured in dprime (**a**), hit rate (**b**), and false alarm (**c**) for both emotional (striped) and neutral (white) words in young and older adults. Error bars represent SEM

We first examined the impact of age and sleep on recognition memory performance. We did not find a main effect of sleep on recognition memory, dprime: *F*(1,208) = 1.13, *p* = .29, *η*^2^ = .006, hit rate: *F*(1,208) = 0.53, *p* = .46, *η*^2^ = .003, false alarm: *F*(1,208) = 3.76, *p* = .054, *η*^2^ = .02. We found a significant main effect of age on all memory indicators, with young adults showing better recognition performance, dprime: *F*(1,208) = 27.64; *p* < .001, *η*^2^ = .12; hit rate: *F*(1,208) = 22.34, *p* < .001, *η*^2^ = .10, and less false alarms: *F*(1,208) = 12.59; *p* < .001, *η*^2^ = .06. We also found a main effect for word-type on hit rate performance, *F*(1,208) = 5.48, *p* = .02, *η*^2^ = .02, such that memory was higher for negative, compared to neutral word pairs. This negative-bias was not present in dprime, *F*(1,208) = 0.45, *p* = .50, *η*^2^ = .002,or in false alarms, *F*(1,208) = 0.11, *p* = .74, *η*^2^ = .06. Analyses also revealed a significant interaction for Age×Word-Type on dprime performance, *F*(1,208) = 8.74; *p* = .003, *η*^2^ = .04, and false alarms, *F*(1,208) = 4.42; *p* = .04, *η*^2^ = .02, where younger adults showed better memory for negative word pairs, compared to neutral word pairs, dprime: *F*(1,208) = 5.97, *p* = .01; hit rate: *F*(1,208) = 4.07, *p* = .04; false alarm: *F*(1,208) = 1.42, *p* = .23 (non-significant). This effect was not present for hit rate, *F*(1,208) = 0.54, *p* = .46, *η*^2^ = .02. In contrast, older adults had fewer false alarms for neutral compared to negative word pairs, indicating decreased memory intrusions, false alarm: *F*(1,208) = 4.03, *p* = .04. No differences in dprime, *F*(1,208) = 2.85, *p* = .09, or hit rate, *F*(1,208) = 1.40, *p* = .23, emerged (see Fig. 3). In addition, no significant interactions were found for Sleep×Age×Word-Type (all *p*s > .17), Sleep×Age (all *p*s > .39), or for Sleep×Word-Type (all *p*s > .18). These results are aligned with previous findings and support tenets of the socioemotional selectivity theory (Carstensen et al., 1999). As memory performance appears to decline with age and younger adults tend to have better memory retention rates for negatively valenced information than older adults (Jones et al., 2016).

### Subjective Emotional Reactivity

We found no difference in LTM performance after a nap vs wake. As such, we collapsed across the sleep and wake groups to examine the changes in valence and arousal ratings, using difference scores (session 2 − 1) for younger and older adults. For these data, positive difference scores represent increased positivity in valence and higher levels of arousal at session 2. For arousal, a significant main effect of age emerged such that younger adults reported higher arousal, *F*(1,178) = 6.13; *p* = .01, *η*^2^ = .03. A main effect of valence also emerged as all participants reported higher valence ratings for negative compared to neutral word pairs, *F*(1,178) = 7.10; *p* = .008, *η*^2^ = .04. No age differences emerged for valence, *F*(1,178) = 0.11; *p* = .74, *η*^2^ = .001, indicating that younger and older adults did not differ in the positive or negative attributions given to the negative and neutral words presented. Additionally, no word-type differences were present for arousal, *F*(1,178) = 0.22; *p* = .64, *η*^2^ = .001, in that negative or neutral words did not differ significantly in their subjective arousal ratings. We did find a significant interaction for Age×Word-Type for valence, *F*(1,178) = 5.81; *p* = .02; *η*^2^ = .04, such that older adults rated neutral and negative words more positive across the day, this difference was not present for younger adults. We did not find an Age×Word-Type interaction for arousal, *F*(1,178) = 0.06; *p* = .81, *η*^2^ = .001.

Using one-sample *t*-tests, we compared both valence and arousal scores to zero (no change) and found no significant change in ratings for both negative, valence: *t*(52) = 0.62, *p* = .54; arousal: *t*(52) = 1.20, *p* = .24, and neutral word pairs, valence: *t*(34) = 0.33, *p* = .74, arousal: *t*(34) = 1.66, *p* = .10, in young adults. On the other hand, older adults rated word pairs more positively across the day for both negative, valence: *t*(47) = 2.44, *p* = .02, and neutral word pairs, valence: *t*(49) = 2.12, *p* = .04. No differences in arousal were present, negative: arousal: *t*(47) = −1.61, *p* = .11 (non-significant); neutral: arousal: *t*(49) = −1.29, *p* = .20 (non-significant). These results indicate that along with a positivity bias in memory, older adults also show greater positivity in subjective ratings of valence across the day, whereas younger adults remained stable in their ratings.

### LTM and Emotional Reactivity Association

Next, we examined the associations between the change in valence with LTM performance. In younger adults, we found no significant associations between recognition and change in valence in either neutral (all *p*s > .39) or negative word pairs (all *p*s > .54). However, in older adults, change in valence across the day was related to greater neutral memory performance, dprime: *r* = .31, *p* = .03; false alarm: *r* = −.28, *p* = .045; hit rate = .26, *p* = .06 (trend). In addition, among older adults, change in valence of negative words was related to higher dprime (*r* = .37, *p* = .009) but not to false alarm or hit rate (all *p*s > .14). No correlations were present for arousal (all *p*s > .27). These results indicate that increased positivity may support better memory in older adults. Taken together with the positivity bias in memory and the greater positivity in subjective ratings of valence across the day, for older adults, the associations between emotion and LTM may indicate an age-specific memory strategy and provide new evidence for the socioemotional selectivity theory.

### LTM and WM Associations

**Fig. 4:**
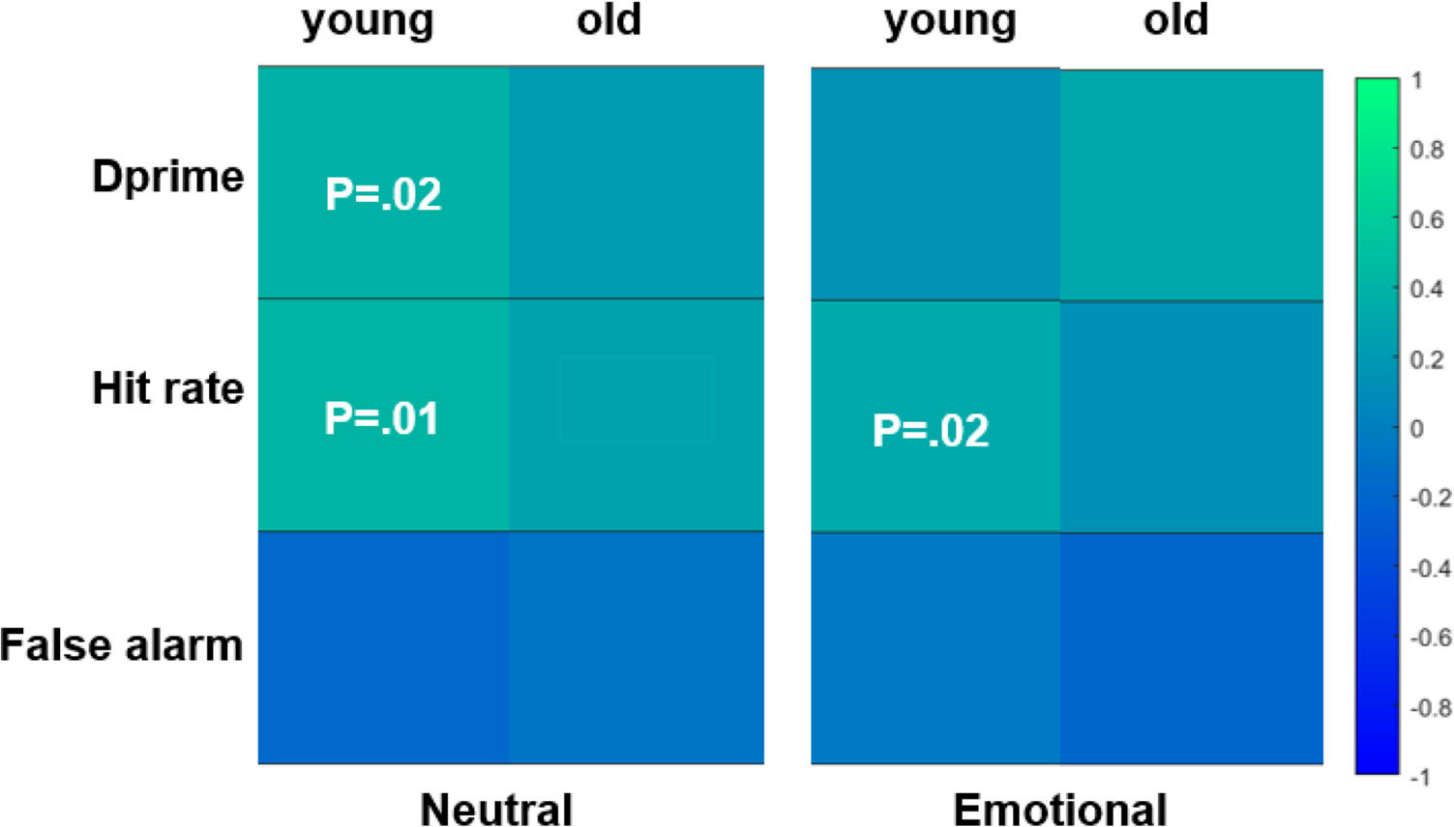
WM and LTM (dprime, hit rate, and false alarm) associations in young and old adults for neutral (left) and emotional (right) word types. Color represents the effect size *r* with blue shows negative, and green shows positive associations. *p*-value is reported for significant associations (*p* < .05)

Figure 4 presents the effect size *r* for the correlations between WM and dprime, false alarm, and hit rate of the WPA performance by age and word-type. In younger adults, better WM performance was related to higher hit rate (*r* = 0.39, *p* = .01) and dprime (*r* = .38, *p* = .02) for neutral word pairs, and higher hit rate for negative word pairs (*r* = 0.34, *p* = .02; see Fig. 4). However, in older adults, we did not find significant associations between WM and WPA performance (all *p*s > .08). Additionally, no significant associations were found between WM and valence or arousal for any of the word pairs in older or younger adults (all *p*s > 0.07). These results suggest that WM and LTM may share related processes (Ranganath et al., 2003), yet the robustness of this relationship may fade with age (see Supplement 1 for the scatterplots).

## Discussion

Our study sought to measure the contribution of working memory, emotional salience, and long-term memory maintenance across a period of sleep or wake in younger and older adults. Similar to prior results, younger adults had less fragmented sleep and received more slow wave sleep and REM and less light stage 1 sleep compared to older adults. However, the presence or absence of sleep did not affect recognition memory in either age group. Younger adults had better overall memory compared to older adults and showed a negativity bias with greater memory for negative pairs compared to neutral, whereas older adults showed better memory for neutral pairs compared to negative. In younger adults, emotional reactivity, measured by the change in valence, remained stable over time and did not predict later memory. However, older adults showed decreased negativity that was correlated with better LTM. Further, WM showed positive associations with LTM in younger, but not older adults, suggesting that there may be overlapping benefits afforded to both LTM and WM domains that may fade with aging. In summary, we show that age-related decline in the retention of negative long-term memories may be associated with age-related changes in WM dynamics and a shift from negative to positive associations with learned experiences. These findings provide further evidence of age-related shifts in perspective and motivated emotional well-being goals shaping long-term memory retention for older adults (Carstensen et al., 1999).

LTM refers to the ability to hold information in mind indefinitely (Cowan, 2008). Importantly, however, changes to cognitive and physiological factors across aging shift what information may be held indefinitely. In our study, the younger adults showed better memory for negatively valenced word pairs, whereas older individuals showed worse memory for these same negative word pairs, indicating that the strength of the association between memory items depends on the emotional salience of the words. This adds to the existing evidence supporting the socioemotional selectivity theory (SST; Carstensen, 2006; Carstensen et al., 1999), which posits that the perception of the future can impact the relative importance placed on different types of goals. This theory hypothesizes that young adults may perceive their futures to be relatively open-ended, and therefore prioritize learning and novel experiences, which may include valuing negative vs neutral or positive experiences. Alternatively, older adults, who perceive their futures to be relatively limited, prioritize well-being and as such are biased to remember positive events. Consistent with SST hypothesis, our older adults showed lower performance for negative memories.

These data also suggest that WM may be an important determinant of several higher-order cognitive functions (Engle, 2002; Borella et al., 2010; Nettelbeck & Burns, 2010), including LTM (Burgess & Hitch, 2005). Similar to other cognitive domains, WM capacity declines with age (Wingfield & Tun, 2002) and it has been shown that WM and LTM are more tightly associated among younger adults (Lugtmeijer et al., 2019; Yonelinas, 2013). Lugtmeijer et al. (2019) tested 30 younger and 30 older adults on an N-back WM task and an object location LTM task and reported that, compared to younger adults, older individuals had a decline in both WM and LTM performance. In addition, performance in the WM and LTM tasks were correlated among younger, but not older adults. Similarly, we found that indeed WM was related to better LTM performance for both emotional and neutral word pairs among younger adults and this was not true of older adults. In older adults, we found a reduction in LTM, compared to younger adults, and no relation between WM and LTM performance regardless of the emotional component of the word pairs. This contrasts with Mather and Knight (2005) who reported an age-related positivity bias for older adults where higher WM performance among older individuals predicted better memory for positively valenced pictures in comparison to negatively valenced stimuli. One reason for this discrepancy may be the difference in stimuli type as Mather and Knight (2005) used valenced pictures and we used valenced word pairs. Another concern may be the associative nature of the word pair stimuli we engaged in our study compared to the item like memory of the pictures in Mather and Knight (2005).

### Limitation

A number of limitations impacted the results of this study. First, the data in this study were part of a study designed to examine how sleep impacts ERPs important for working and long-term memory across a day in older and young adults. As such, methodological choices aligned with the ERP design precluded the ability to utilize an immediate test for the memory tasks. Therefore, we were unable to measure the amount of change in memory across the day. Future studies should explore memory change across sleep vs wake to better assess the role of sleep on these dynamics. Additionally, given the original study was designed with ERP features important for working and long-term memory, we did not conduct a power analysis for the specific outcome variables highlighted in the current manuscript, which may make us underpowered to detect some of the effects. Importantly, however, the sample sizes that we included in the current study are aligned with other projects exploring the effect of WM, emotionality, and sleep on LTM outcomes (Mather & Knight 2005; Jones et al., 2016).

In conclusion, we found an emotional bias in both memory and emotional reactivity, with decreased emotional reactivity associated with better memory in older adults, and a robust association between WM and LTM in younger but not older adults. These effects did not differ dependent on a daytime nap. Our findings are consistent with the socioemotional selectivity theory that posits that aging is associated with a relative suppression of negative information, suggesting that this bias is more related to the saliency of the stimuli and motivation to preserving well-being rather than with WM processes. In contrast, robust WM functioning may play an important role in processing LTM in young adults, a relationship that may fade with decreased WM processing as we age.

Finally, these findings may provide some evidence into how mechanistic changes associated with aging, specifically working memory and emotional saliency support (or fail to support) memory functions. One important future investigation would be to explore the temporal relationship between working memory and emotional saliency changes. In context of the current findings, it would be valuable to know which factor changes first, salient motivational goals to maintain well-being or working memory shifts, as that would help further illuminate aging-related changes in cognitive function. On the other hand, working memory training protocols have been shown to improve working memory over multiple days of training, with generalization of the benefits to long-term memory across both younger and older adults (see meta-analysis Au J, Sheehan E, Tsai N, Duncan GJ, Buschkuehl M & Jaeggi SM, 2015; Karbach & Verhaeghen, 2014). Future investigations exploring the association between emotional saliency, working memory, and long-term memory in older adults, in the memory training context, are warranted.

## Supplementary Information

ESM 1 (DOCX 161 kb)

## Additional Information

### Data Availability

Data for this study can be found here: https://osf.io/6bvmw/
