# Supplementary material for: The Role of Working Memory in Age-Related Emotional Memory Bias
Source: Affect Sci. 2022 Aug 31;3(3):686–95. doi: 10.1007/s42761-022-00134-5 (PMC9537401; doi:10.1007/s42761-022-00134-5)
Supplement: Supplementary file 1 — (DOCX 161 kb) [file 42761_2022_134_MOESM1_ESM.docx]

**Supplement:**

**Figure 1 and 2 here represents the scatterplots, identical to the correlations presented in the colored table in the manuscript.**


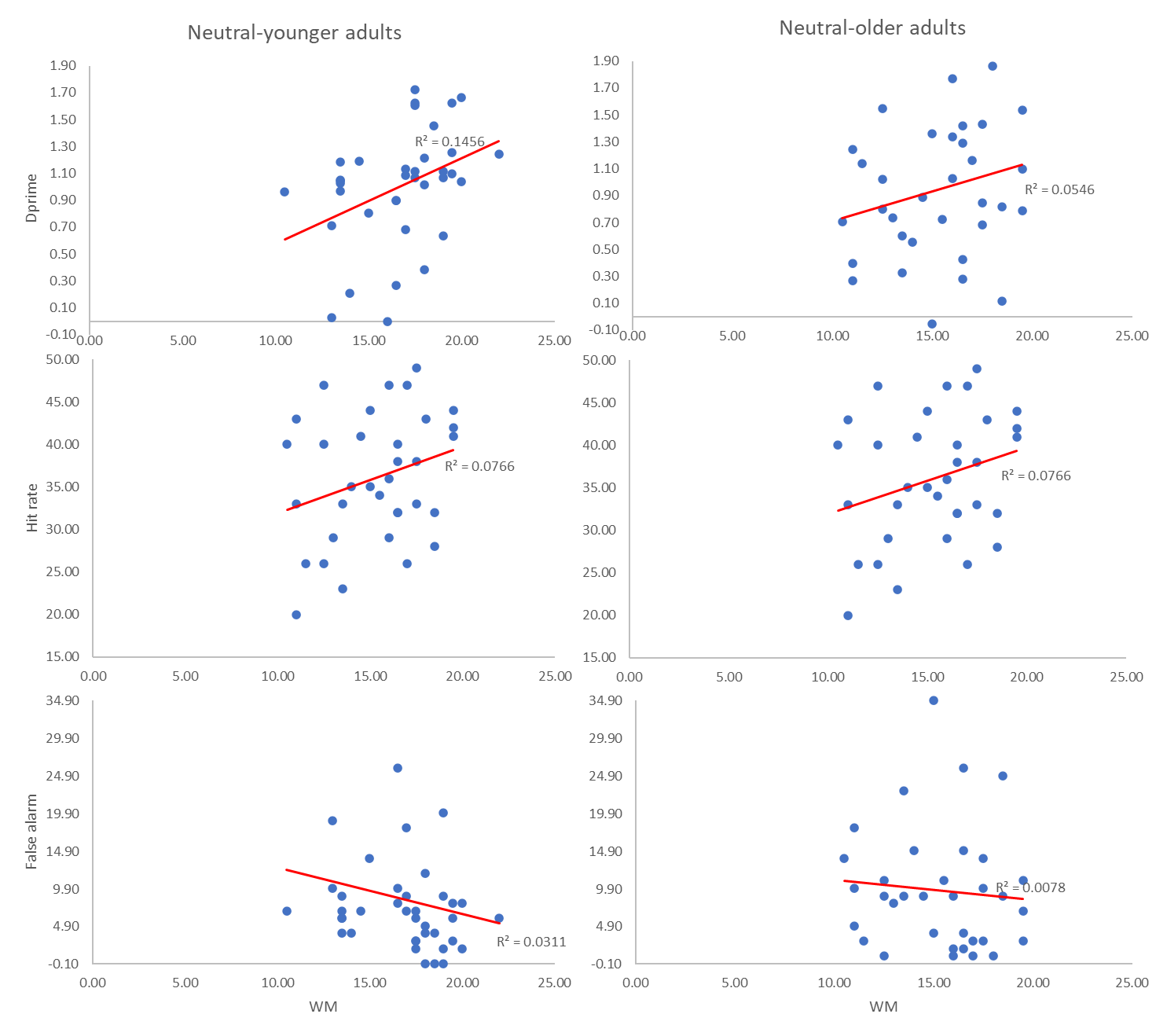


Figure 1: WM and LTM [dprime(first row), hit rate (second row) and false alarm (third row)] associations in Young (left pannels) and Old (right pannels) adults for neutral word types.


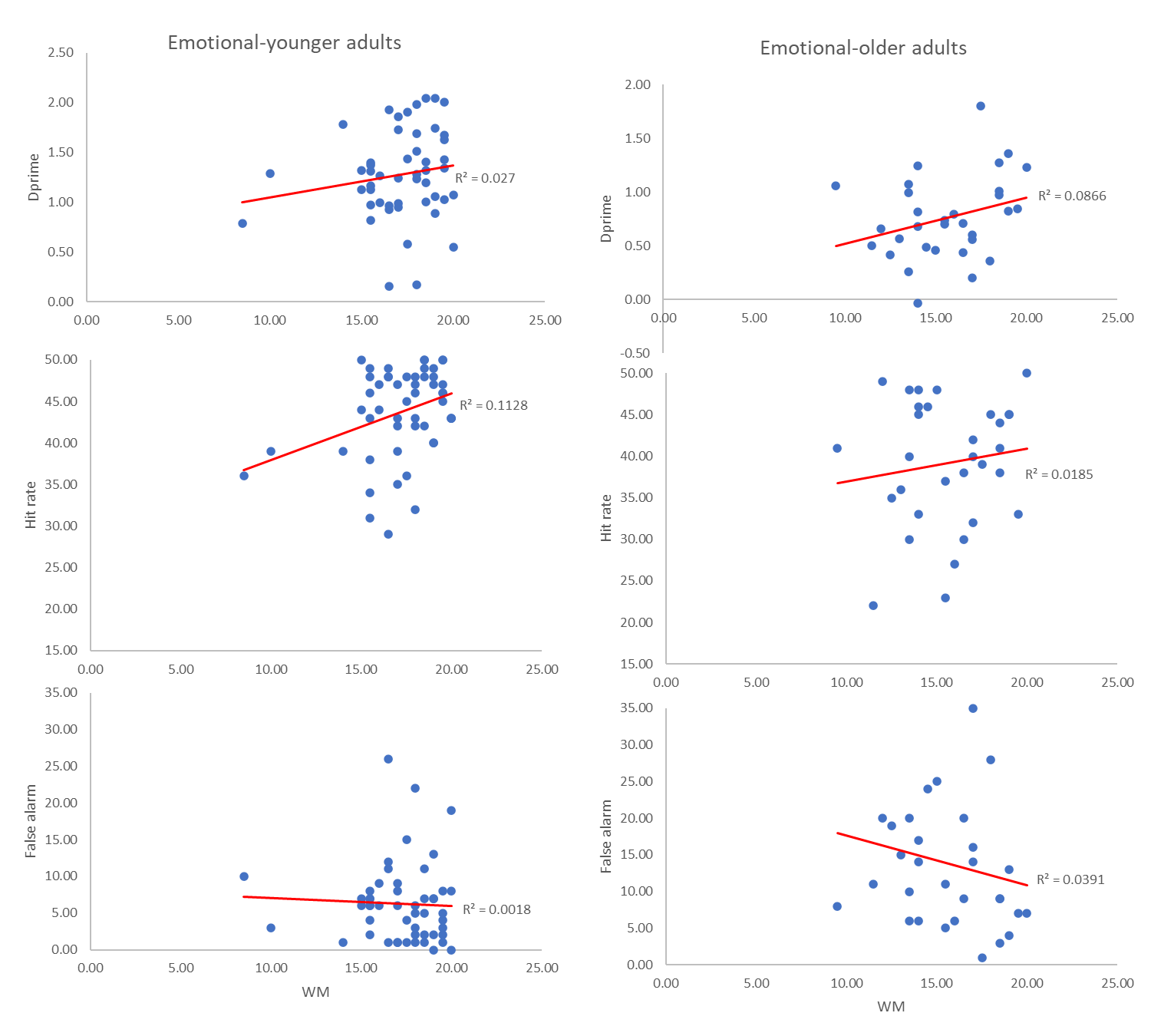


Figure 2: WM and LTM [dprime(first row), hit rate (second row) and false alarm (third row)] associations in Young (left pannels) and Old (right pannels) adults for emotional word types.
